# HyenaCircle: a HyenaDNA-based pretrained large language model for long eccDNA prediction

**DOI:** 10.3389/fgene.2025.1641162

**Published:** 2025-06-26

**Authors:** Fuyu Li, Wenxiang Lu, Yunfei Bai

**Affiliations:** State Key Laboratory of Digital Medical Engineering, School of Biological Science and Medical Engineering, Southeast University, Nanjing, China

**Keywords:** extrachromosomal circular DNA, eccDNA, large language model, deep learning, long eccDNA prediction, third-generation sequencing

## Abstract

**Introduction:**

Extrachromosomal circular DNA (eccDNA) represents a class of circular DNA molecules derived from chromosomes with diverse roles in disease. Long eccDNAs (typically 1–5 kb) pose detection challenges due to their large size, hindering functional studies. We propose HyenaCircle, a novel deep learning model leveraging large language model and third-generation sequencing data to predict long eccDNA formation.

**Methods:**

Full-length eccDNAs within 1–5 kb were identified by FLED algorithm for Nanopore sequencing data, extended by 100-bp flanking sequences, and paired with 20,000 length-matched negative controls from eccDNA-depleted genomic regions. HyenaCircle was built by adapting the pretrained HyenaDNA model with a designed classifier head. The strategies of data augmentation, regularization and class imbalance weighting were applied to increase model robustness.

**Results:**

HyenaCircle achieved comparable performance with a validation AUROC of 0.715 and recall of 0.776. It surpassed DNABERT by 5.9% in AUROC and demonstrated stable convergence. Hyperparameter optimization confirmed batch size 16 and learning rate 5 × 10^−5^ as optimal. The ablation studies revealed flanking sequences are important, as their removal reduced model stability. The model also showed superior stability over the baseline HyenaDNA architecture.

**Conclusion:**

HyenaCircle integrated third-generation sequencing data and large language model for long eccDNA prediction, which outperformed the existing model. Our work demonstrates that the HyenaDNA architecture enables effective long-sequence genomic modeling and provides a new insight for eccDNA prediction and identification.

## 1 Introduction

Extrachromosomal circular DNA (eccDNA) is a distinctive category of DNA molecules characterized by a ring-like structure, formed by the cyclisation of DNA fragments shed from chromosomes (Gaubatz, 1990; Paulsen et al., 2018). The advent of high-throughput sequencing technology has catalyzed the research of eccDNA. It is widely accepted that eccDNA is a significant indicator of genomic instability and eccDNAs with different sizes have significant differences in the formation mechanism, biological properties, relative abundance, and the manifestation in diseases.

In terms of the formation mechanisms, short eccDNAs (less than 1,000 bases) primarily rely on microhomology-mediated repair following DNA double-strand breaks, exhibiting a high degree of stochasticity (Pau et al., 2021; Yang et al., 2022). Conversely, the formation of long eccDNAs (more than 1,000 bases) involves complex events, such as chromosome fragmentation, break-fusion-bridging loops, and eccDNA fusions, and may result in reintegration into the chromosomal genome (Noer et al., 2022; Koche et al., 2020; Eugen-Olsen et al., 2025). Accordingly, with regard to biological characteristics, short eccDNAs typically function as gene regulatory elements, carrying enhancer, promoter, or non-coding RNA sequences that influence local chromatin status or stimulate immune activation through cis-regulation and its circular structure (Wang et al., 2021; Ling et al., 2021). It has been established that long eccDNAs, which have been shown to exhibit the characteristics of a mini genome, contain a complete set of oncogenes and their regulatory elements. These eccDNA-encoded genes, such as *MYCN* and *CCND1*, undergo amplification and heterogeneous distribution through the independent replication units present on eccDNAs (Helmsauer et al., 2020; Ambros et al., 1997). In normal tissues, eccDNA molecules are predominantly constituted of short fragments, with a proportion exceeding 90% measuring less than 1 kilobase. Conversely, tumor tissues exhibit a significantly higher abundance of long eccDNA (Yang et al., 2022; Møller et al., 2018). Consequently, in comparison with short eccDNA, long eccDNA is often deemed to possess a higher clinical value, particularly in the context of complex diseases such as malignant tumors. In glioblastoma, long eccDNAs carrying *EGFR* amplicons form a super-enhancer structure, driving the sustained activation of oncogenes and thus influencing the tumor progression (Wu et al., 2019; Morton et al., 2019; Zhu et al., 2021; Karami et al., 2022; Nikolaev et al., 2014). In colorectal cancer, long eccDNA carrying genes such as *DHFR* and *EGFR* induces the drug resistance in tumor cells (Morales et al., 2009; Morales et al., 2005; Ooi et al., 2004; Chen et al., 2023); while in neuroblastoma, patients carrying *MYCN*-amplified eccDNA show an even lower survival rate (Karami et al., 2022; Nathanson et al., 2014; Chapman et al., 2023). It has been demonstrated that eccDNA is significantly more stable than linear DNA in the tumor microenvironment, and its circular structure resists degradation by DNA exonucleases. This renders eccDNA an ideal target for liquid biopsy (Ling et al., 2021; Luo et al., 2023; Ya et al., 2024; Lv et al., 2022). It is therefore imperative to identify and parse long eccDNA accurately and efficiently.

Current researches on the eccDNA identification and characterization are mainly based on traditional bioinformatics approaches. Various algorithms have been designed to identify the unique molecular signature of eccDNA based on the alignment of sequencing reads. For instance, classic tools such as Circle_finder and Circle-Map, infer potential circular structures by identifying split and discordant alignment patterns of sequencing reads, specifically soft clipped reads and discordant aligned read pairs, while enhancing prediction accuracy through the analysis of local sequencing depth variations and statistical validation around the joint breakpoints (Shibata et al., 2012; Dillon et al., 2015; Prada-Luengo et al., 2019; Prada-Luengo et al., 2020). In contrast, methods for eccDNA identification based on third-generation sequencing (TGS) data, such as Flec and FLED, leverage the ultra-long read lengths characteristic of TGS to precisely identify eccDNA molecules and reconstruct their internal structures and full-length sequences (Wang et al., 2021; Li et al., 2023; Wang et al., 2023). However, existing methodologies remain heavily dependent on specific experimental protocols and sequencing technologies, and face challenges in detecting long eccDNA due to inherent limitations imposed by DNA polymerases and achievable read lengths. AmpliconArchitect and AmpliconReconstructor reconstruct the internal structure and sequences of long eccDNAs by analyzing abnormal alignment patterns and copy number variations caused by eccDNAs in whole-genome sequencing data, or combined with optical mapping data. Recently, a machine learning model, GCAP, developed by Zhao et al. leverages whole-exome sequencing data to predict long eccDNA amplifications and associated genes in tumor from the perspective of genomic copy number variation (Wang et al., 2024). Although this approach partially addresses limitations inherent in traditional alignment-based methods, the reliability of GCAP predictions is contingent upon accurate copy number profiles. And due to the typically low copy numbers of long eccDNAs, AmpliconArchitect, AmpliconReconstructor and GCAP struggles to reconstruct complex eccDNA structural and consequently fails to resolve the intrinsic complex sequence of eccDNAs. These limitations result in constrained prediction accuracy and generalization ability. Consequently, comprehensively extracting the implicit intrinsic features from eccDNA sequences while maintaining the integrity poses a significant challenge in current research.

Transformer-based large language models (LLMs) have demonstrated revolutionary potential in genomics. Through self-supervised pre-training strategies, LLMs can learn deep grammatical rules of DNA from billions of base pairs, enabling breakthroughs in predicting cis-regulatory elements (Le et al., 2022; Luo et al., 2022), identifying splice sites (Ji et al., 2021), characterizing DNA-protein interactions (An et al., 2024; An et al., 2022), and forecasting DNA methylation (Tsukiyama et al., 2022; Jin et al., 2022), thereby overcoming limitations inherent in traditional feature engineering. Representative models like DNABERT, which employ k-mer tokenization strategies and undergo pre-training on the human reference genome, achieve state-of-the-art (SOTA) performance in tasks such as promoter prediction, transcription factor binding site identification, and splice site recognition with minimal fine-tuning (Ji et al., 2021). Notably, enhanced long-sequence modeling capabilities of LLMs present new opportunities for eccDNA research. Moreover, compared to traditional bioinformatics methods such as FLED and Circle-Map, this data-driven modeling approach offers distinct advantages: it can uncover previously unknown or non-genomic-origin eccDNA sequence patterns and quantitatively characterize eccDNA sequence features, thereby facilitating the effective integration of multi-omics data.

Traditional transformer attention mechanisms face two primary constraints: first, computational complexity bottlenecks impose sequence length limitations, where increasing sequence length incurs exponential computational resource demands, restricting existing models to input lengths typically between 500 bp and 4 kb—merely 0.01% of the human genome; second, current efficiency-focused models sacrifice base-level resolution, impeding single-nucleotide precision predictions. Emerging architectures like HyenaDNA address these limitations by replacing multi-head attention with Hyena operators (Nguyen et al., 2023). HyenaDNA incorporated implicit long-range convolutions and processed single nucleotide tokens, and pretrained on the human reference genome with context lengths of up to 1 million tokens at the single nucleotide-level, finally achieving SOTA performance. This architectural breakthrough enables direct prediction of long eccDNA formation from long contiguous DNA sequences.

Moreover, previous studies indicate that LLMs can capture key sequence governing eccDNA formation. DeepCircle, a framework integrating DNABERT with convolutional neural networks (CNNs), has established predictive models for short eccDNAs, achieving cross-dataset prediction accuracies of 79.65% (CNN-based) and 83.31% (DNABERT-based) respectively (Chang et al., 2023). It demonstrates the inherent predictability of eccDNA sequence. Nevertheless, current LLM-based eccDNA prediction approaches remain constrained by two primary limitations. First, existing LLMs are predominantly designed and optimized for short eccDNAs, whereas long eccDNAs exhibit more complex structural characteristics and formation mechanisms requiring dedicated long-sequence modeling strategies. Second, training eccDNA sequences primarily derived from NGS data, suffers from short-read limitations that compromise full-length sequence reconstruction of long eccDNAs, consequently impairing model capabilities in capturing structural variations.

To address these challenges, we employed third-generation sequencing (TGS) to accurately identify and sequence full-length eccDNAs, with particular emphasis on long eccDNAs (1–5 kb). Building upon the HyenaDNA architecture, we developed HyenaCircle through model optimization, harnessing its long-range dependency modeling capacity for comprehensive analysis of long eccDNA sequence characteristics. This framework enabled us to establish a predictive algorithm for long eccDNA formation, followed by carefully evaluation of model generalizability. Together, these contributions offer both methodological advances and new perspectives for investigating tumor genomic instability.

## 2 Materials and methods

### 2.1 EccDNA sequencing

In this work, we used seven human cell lines for eccDNA detection, including gastric cancer cells BGC823 and SGC7901, gastric epithelial cells GES1, hepatocellular carcinoma cells HepG2, liver cells HL7702, breast carcinoma cells MDA-MB-453 and breast epithelial cell line MCF12A. Each sample was subjected to Plasmid-Safe ATP-dependent DNase (Epicentre) digestion and performed rolling circle amplification by Phi29 DNA polymerase with Exo-resistant random primer followed by Nanopore sequencing according to the manufacturer’s protocol. High-quality base-called reads were aligned to the human reference genome (GRCh38) using long-read splice-aware mapper minimap2 (Li, 2018; Li, 2021).

### 2.2 Samples and datasets

FLED algorithm was employed to identified high-confident eccDNAs from Nanopore sequencing data in 7 cell lines. FLED is a full-length eccDNA detection methods that was developed in our previous work (Li et al., 2023). Multilevel annotation was then performed using Bedtools (Quinla, 2014; Quinlan and Hall, 2010) intersect (v2.30.0) with gene annotation information from the GENCODE database (GRCh38, Release 45). First, a stringent full-exon coverage criterion, which means that the exons completely contained within eccDNA regions, was applied to select eccDNAs carrying intact exons. Subsequently, the gene-body full-structure coverage criterion, which means the gene body regions fully encompassed by eccDNA regions, was used to define complete genic eccDNAs. Functional annotation was also conducted for eccDNAs overlapping with gene regions.

Based on the length distribution characteristics of eccDNA with gene or exon structures, a dynamic length filtering threshold (min = 1,000 bp, max = 5,000 bp) was established, retaining only qualified eccDNAs across the 7 cell lines. To preserve potential regulatory elements around eccDNA breakpoint regions, such as transcription factor binding sites or open chromatin regions, we extended 100 bp upstream and downstream genomic sequences for each eccDNA using Samtools (v1.15) (Li et al., 2009), to generate continuous sequences as the positive sample dataset used in this work. Specifically, the total length of each positive sample comprised both the full-length eccDNA sequence and 100 bp flanking sequences on each side.

The next step was the generation of random genomic sequences. We first merged all FLED-detected eccDNAs from the 7 cell lines using Bedtools merge (v2.30.0) and identified shared eccDNA-depleted regions across samples using Bedtools complement. The resulting genomic regions totaled 1.9 Gb, where no homologous eccDNAs were detected in any of the 7 cell lines. Subsequently, we employed a stratified sampling approach to match the length distribution of positive samples, for example, the eccDNA regions plus 100 bp flanking sequences on both sides. Using Bedtools random, we dynamically generated a candidate dataset with sequences of equal length to the positive set. Finally, Monte Carlo simulation was applied to optimize spatial uniformity in sampling, yielding a negative control dataset comprising 20,000 sequences. Each negative sequence contained a core region and flanking sequences matching those of positive samples, with total lengths ranging from 1.2 kb to 5.2 kb, which used as the negative sample dataset used in this work.

### 2.3 Datasets partitioning, data augmentation and preprocessing

The positive and negative sample datasets were divided into training and internal validation sets through stratified random partitioning at an 8:2 ratio, ensuring consistent class distribution between both datasets.

To improve model robustness and generalization capability, we employed multiple data augmentation strategies to further expand the dataset. The overall data augmentation approach involved applying dynamic spatial transformations to the training sequences, including the following preprocessing operations. First, a 50% probability of reverse complement conversion was implemented on original sequences to simulate the double-stranded nature of DNA, achieving data augmentation based on the reverse complement principle. Second, for sequences exceeding 500 bp in length, random truncation was performed to retain 50%–100% continuous subsequences as training data, enhancing the model’s sensitivity to local features. Additionally, the model incorporated a 10% probability of random base substitution to simulate potential single-base mutations and sequencing errors. Specifically, this 10% probability operated at the sample sequence level, meaning each training sample had a 10% chance of triggering a base substitution event during augmentation. When triggered, the model would randomly select a single base position in the sequence for substitution, such as replacing the original C with any of A/T/G. This design resulted in an actual per-base mutation probability of 0.1 × (1/L). Considering that FLED employs a multiple sequence alignment strategy to generate consensus sequences during eccDNA reconstruction, the base accuracy of the corrected sequences can be comparable to that of high-throughput sequencing. So for the 5 kb sequences examined in this study, the actual single-base mutation probability was 0.002%. By introducing this sparse mutation strategy, the enhanced sequences maintained error rates on the same order of magnitude as quality-controlled real data, without introducing unnecessary errors or confounding information.

Furthermore, to address potential class imbalance issues, the model adopted a dynamic weight adjustment strategy based on sample size. The weight coefficients were determined by calculating the inverse of class frequencies, with the specific formula as follows:

$$W_{i}=\frac{n_{samples}}{n_{classes}*C_{i}}$$

where $W_{i}$ represents the class-corrected weight coefficient, $n_{samples}$ is the total number of training samples, $n_{classes}$ is the total number of classes, and $C_{i}$ is the actual sample count for class *i*. During model training, these class correction weights were further integrated into the cross-entropy loss function to optimize the loss function.

### 2.4 Construction of HyenaCircle

This work presented some model improvements based on the HyenaDNA architecture, a deep learning framework specifically designed for DNA sequence classification (Nguyen et al., 2023). In contrast to Transformer-based approaches such as DNABERT, HyenaDNA employs a more efficient mechanism for capturing long-range dependencies, enabling superior processing of lengthy sequence data while simultaneously reducing computational and memory requirements. The architecture incorporates a pretraining module for key feature extraction combined with a lightweight classification network, achieving enhanced training and inference speeds while demonstrating greater robustness and practicality in handling the complexities of genomic data.

Due to computational resource constraints, specifically the 24 GB memory limitation of our NVIDIA GeForce RTX 4090D GPU and considering that the eccDNA sequences under investigation do not exceed 6 kb in length, we ultimately adopted the pretrained weights from the hyenadna-small-32k model. This model supports input sequences up to 32 kbp in length, with pretrained parameters available at: https://huggingface.co/LongSafari/hyenadna-small-32k-seqlen/tree/main.

**FIGURE 1:**
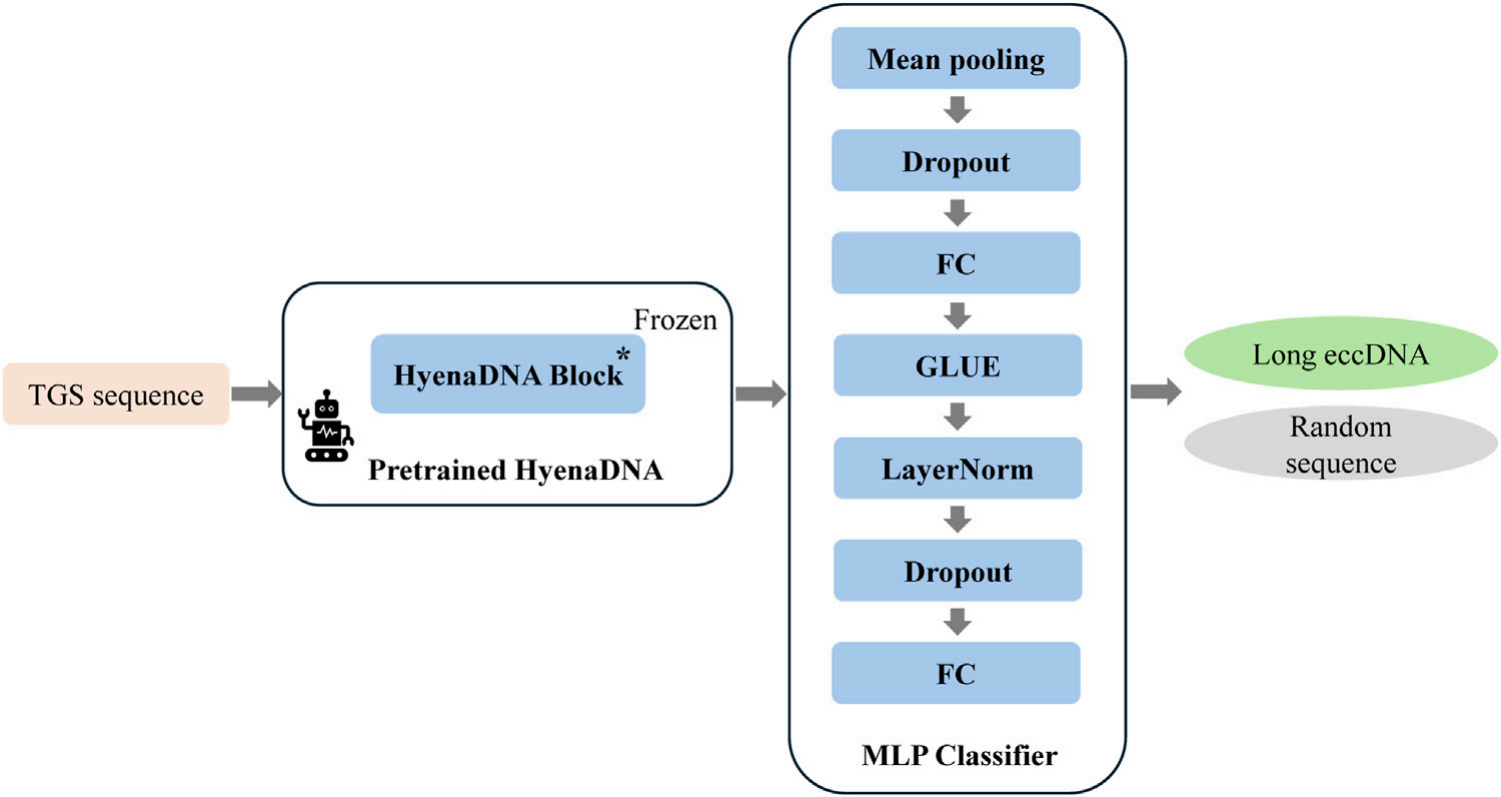
Network architecture diagram of the HyenaCircle model.

To develop an AI model for long eccDNA identification based on sequence information, we implemented targeted optimizations based on the HyenaDNA architecture, resulting in our proposed HyenaCircle model. The implementation process begins with tokenization of full-length eccDNA sequences prior to model construction. To maintain single-nucleotide resolution, HyenaCircle directly utilizes the four nucleotide bases along with necessary special characters as its vocabulary, converting sequences into numerical vectors for model input. Specifically, the HyenaCircle retains the frozen HyenaDNA block from the pretrained model as its feature extraction backbone, maintaining an output dimensionality of 768. For the classification component, the architecture incorporates an adaptive average pooling layer to reduce sequence dimensionality, followed by a feature transformation module consisting of a 512-dimensional fully connected layer, GeLU activation function, layer normalization, and dropout (rate = 0.3), ultimately producing binary classification outputs through a linear projection layer. The network architecture of our proposed HyenaCircle model is illustrated Figure 1.

During the training phase, HyenaCircle was trained by using a mixed-precision training strategy that employed the bfloat16 floating-point format to accelerate computational processes. The AdamW optimizer was implemented with an initial learning rate of 4 × 10^−5^, coupled with a cosine annealing scheduler that incorporated a linear warm-up period during the first 10% training steps to prevent gradient instability. To mitigate overfitting, the network incorporated multiple regularization mechanisms. A weight decay coefficient of 0.01 was applied to constrain the parameter space, while gradient clipping with a maximum norm of 1 was implemented to control gradient magnitudes. Label smoothing was integrated into the loss function to enhance model calibration capabilities.

The training process was conducted on an NVIDIA GeForce RTX 4090D GPU (24 GB VRAM), with gradient accumulation employed to alleviate memory constraints (accumulation steps = 2). Under these configurations, each complete training epoch requires approximately 6 h on average. This optimization strategy effectively balanced computational efficiency with model performance while working within hardware limitations.

### 2.5 Model performance evaluation and optimization

Given the current paucity of research attempting to establish predictive models for long eccDNA formation based on sequence information, we implemented a comprehensive and objective evaluation framework to assess model performance and the feasibility of this work. Our multi-dimensional evaluation system incorporated six key metrics: area under the receiver operating characteristic (AUROC), specificity, accuracy, precision, recall, and F1-score, providing a robust assessment of the model’s classification performance, with specificity = TN/(TN + FP), accuracy = (TP + TN)/(TP + TN + FP + FN), precision = TP/(TP + FP), recall = TP/(TP + FN), and F1 = 2 × Precision × Recall/(Precision + Recall); where TP denotes true positives, TN denotes true negatives, FP denotes false positives, and FN denotes false negatives. Throughout model training and validation, the dataset was randomly partitioned into training and independent test sets at an 8:2 ratio to prevent data leakage.

For critical hyperparameters including batch size and learning rate, we systematically determined the optimal combination through the following approach. To balance training efficiency with memory constraints, we evaluated four batch size options (Yang et al., 2022; Wang et al., 2021; Karami et al., 2022; Wang et al., 2023). The learning rate optimization in this work was conducted in two phases: an initial coarse search across four values based on previous experience (1 × 10^−4^, 3 × 10^−4^, 1 × 10^−5^, 5 × 10^−5^) followed by a refined search in the vicinity of the optimal learning rate identified during model training. The entire training process comprised 50 epochs, with both training and validation loss functions meticulously recorded and visualized to monitor model convergence and performance. This rigorous parameter optimization strategy ensured robust model performance while maintaining computational feasibility.

### 2.6 Ablation and comparative experiments

To comprehensively evaluate the performance of our proposed HyenaCircle model, we designed two ablation studies: (Gaubatz, 1990): a comparative performance analysis between HyenaCircle and the original hyenadna-small-32k model, and (Paulsen et al., 2018) an assessment of the impact of eccDNA breakpoint flanking sequences on prediction outcomes.

In the first comparative experiment, we focused on optimizing critical hyperparameters (batch size and learning rate) specifically for HyenaCircle during its training process, given their significant relationship with input sequence length. After identifying the optimal hyperparameter combination, we applied the same configuration to train the original hyenadna-small-32k model and evaluated its performance variation. This experiment aimed to assess the feature representation capability of the large model as a feature extractor and compare the performance differences between our classification module integration versus direct model training, providing insights for future network and module design.

The second experiment investigated the influence of eccDNA breakpoint flanking sequences on prediction accuracy. Recognizing that sequences adjacent to eccDNA breakpoints have been demonstrated to correlate with full-length eccDNA identification, we systematically evaluated model performance when varying lengths of these flanking regions were removed. Specifically, we conducted 10 comparative experiments by excluding 10–100 bp (in 10 bp steps) of sequence data from both sides of the breakpoints in the input to HyenaCircle. This allowed us to quantitatively assess the contribution of breakpoint-adjacent sequences to eccDNA formation prediction.

The implementation was conducted in Python 3.10.8 with CUDA 12.1 and PyTorch 2.4.1. Key dependencies included: bio (1.6.2), huggingface-hub (0.29.1), hydra-core (1.3.2), scikit-learn (1.3.2), torchvision (0.20.0), and transformers (4.26.1). This experimental setup ensured reproducibility while leveraging state-of-the-art deep learning frameworks for genomic sequence analysis.

## 3 Results

### 3.1 Genomic feature analysis of positive and negative samples

**FIGURE 2:**
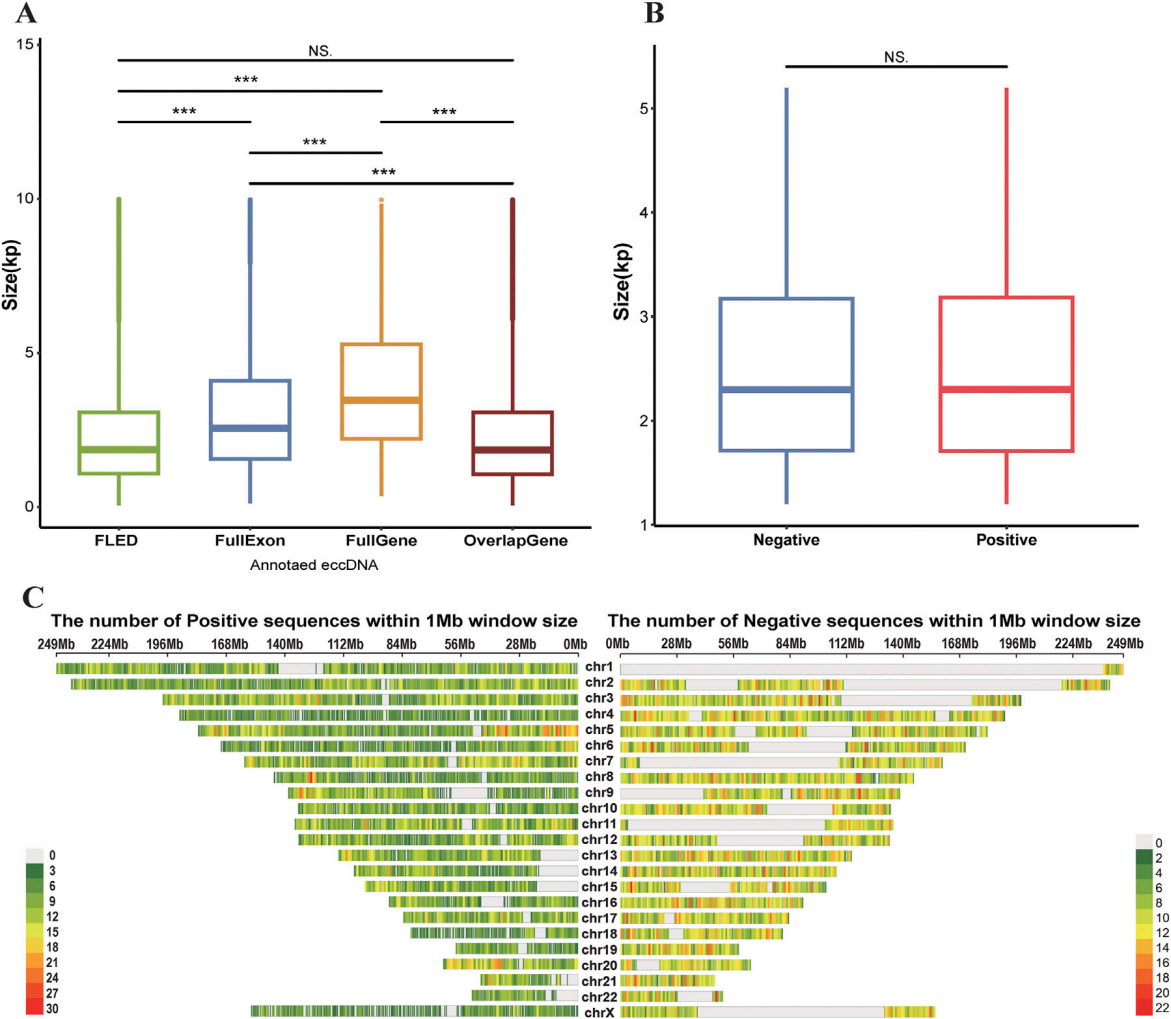
**(A)** The box plot of the size distributions of four eccDNA categories across 7 cell lines detected by FLED: all FLED-identified eccDNAs (FLED, *n* = 45,355), exon-carried eccDNAs (FullExon, *n* = 8,657), eccDNAs with full gene bodies (FullGene, *n* = 1,144), and gene-overlapping eccDNAs (OverlapGene, *n* = 27,922). **(B)** Comparative size distribution of length-filtered eccDNAs (Positive, *n* = 23,812) versus randomly selected genomic sequences (Negative, *n* = 20,000). **(C)** The genomic distribution of length-filtered eccDNAs (Positive, *n* = 23,812) and randomly selected genomic sequences (Negative, *n* = 20,000) across 1 Mb chromosomal windows. P-values are determined using the Wilcoxon test. Significance: (***) P-value<0.001 (NS.) Non-Significant.

Given the widespread genomic distribution of eccDNAs, we first evaluated the representativeness of our modeling dataset by analyzing the genomic features of positive samples, including sequence length distribution and chromosomal distribution. The FLED algorithm identified 45,355 eccDNAs across 7 cell lines using Nanopore sequencing data (Table 1). Based on the eccDNA annotation, these eccDNAs were classified into distinct categories: 8,657 containing complete exons, 1,144 encompassing intact genes, and 27,922 overlapping with gene regions. Length distribution analysis revealed that detected full-length eccDNAs predominantly clustered within the 1–3 kb range, while those carrying intact genes or exons typically spanned 2–5 kb and 2–4 kb, respectively (Figure 2A). Since gene or exon-containing eccDNAs are more likely to possess transcriptional or regulatory potential, we retained 23,812 eccDNAs within the size range of 1–5 kb, along with their flanking genomic sequences as our positive sequence set for subsequent analysis.

**TABLE 1:** Summary of Nanopore sequencing and eccDNA detected by FLED.

Cell line	Number of reads	Total bases	Mean read quality	Read length (N50)	Percentage of clean data (>Q7)	Number of FLED-detected eccDNA	Number of positive eccDNA
BGC823	1,239,061	2,095,226,792	13.7	2,565	98.8%	5,285	2,361
SGC7901	738,344	1,601,836,701	13.7	3,338	98.9%	4,943	2,456
GES1	673,160	1,310,252,216	13.8	3,091	99.1%	6,318	2,701
HepG2	524,970	1,232,425,802	13.9	3,808	99.1%	5,286	3,004
HL7702	2,118,440	3,413,043,155	13.9	2,620	99.0%	3,222	1,218
MB453	1,268,384	2,346,612,631	13.2	3,327	98.6%	19,766	12,198
MCF12A	871,873	1,673,085,152	13.9	3,330	99.2%	535	193

To ensure balanced model training, we randomly selected 20,000 genomic sequences from eccDNA-depleted regions as negative controls, ensuring their length distribution was consistent with that of eccDNAs and their flanking regions. Validation confirmed that 19,719 (98.6%) of the genomic regions of negative samples had an average sequencing depth of less than 2 reads, and the negative sequences exhibited comparable length distribution to the positive set while maintaining uniform genomic distribution (Figures 2B,C). Additionally, we performed a statistical analysis of gene structures encoded in both positive and negative samples: 67.06% of positive sample originated from genic regions, compared to 59.89% in the negative samples. Chi-square test results indicated no significant difference in gene sequence patterns between the positive and negative sets.

### 3.2 Model performance optimization

After finalizing the architecture of HyenaCircle, we optimized two critical hyperparameters, batch size and learning rate, to determine the optimal model configuration. This evaluation aimed to assess their impact on prediction performance for similar tasks while providing practical recommendations for comparable deep learning applications. We systematically tested combinations of batch sizes (Yang et al., 2022; Wang et al., 2021; Karami et al., 2022; Karami et al., 2022; Wang et al., 2023) and learning rates (0.00001, 0.00005, 0.0001, and 0.0003), with performance evaluated across six metrics (Table 2). The results demonstrated significant variations in model performance depending on these hyperparameters.

**TABLE 2:** Results of the HyenaCircle model across multiple performance metrics on the validation set under varying batch sizes and learning rates.

Batch size	Learning rate	AUROC	Accuracy	Precision	Recall	Specificity	F1-score
4	0.00001	0.567	0.551	0.582	0.616	0.474	0.599
4	0.00005	0.713	0.665	0.674	0.742	0.573	0.706
4	0.0001	0.714	0.672	0.667	0.791	0.530	0.724
4	0.0003	0.712	0.670	0.670	0.777	0.543	0.719
8	0.00001	0.599	0.585	0.613	0.641	0.518	0.627
8	0.00005	0.713	0.676	0.660	0.836	0.487	0.737
8	0.0001	0.720	0.679	0.663	0.831	0.497	0.738
8	0.0003	0.714	0.672	0.668	0.789	0.533	0.724
16	0.00001	0.592	0.581	0.607	0.650	0.499	0.628
16	0.00005	0.715	0.670	0.670	0.776	0.545	0.719
16	0.0001	0.712	0.668	0.672	0.759	0.560	0.713
16	0.0003	0.716	0.676	0.669	0.802	0.527	0.729
32	0.00001	0.578	0.562	0.587	0.655	0.451	0.619
32	0.00005	0.712	0.673	0.659	0.824	0.493	0.733
32	0.0001	0.720	0.674	0.675	0.772	0.558	0.720
32	0.0003	0.711	0.668	0.660	0.805	0.505	0.725

Smaller batch sizes (4 or 8) increased parameter update frequency and gradient stochasticity, enhancing model sensitivity but compromising specificity. For instance, at a batch size of eight and learning rate of 0.0001, the model achieved high recall (0.831) and F1-score (0.738), indicating effective feature detection. However, this came at the cost of reduced specificity (0.497) and elevated false-positive rates. In contrast, larger batches (e.g., 32) improved memory efficiency and per-epoch training speed but yielded overly smooth gradient estimates, hindering escape from local optima. At a low learning rate (0.00001), this combination performed poorly (AUROC: 0.578), suggesting ineffective convergence. Even at a learning rate of 0.00005, specificity remained suboptimal (0.545 for batch size 16 vs. lower values for batch size 32), likely due to oversimplified gradient averaging in high-batch settings. Consequently, we discontinued further evaluation of larger batches.

Moderate batch sizes, such as 16, exhibited more balanced performance across learning rates. For example, at a learning rate of 0.00005, this configuration achieved an AUROC of 0.715, F1-score of 0.719, recall of 0.776, and specificity of 0.545, a 12% specificity improvement over smaller batches. While the recall was moderate, the gains in specificity better aligned with practical needs to minimize false positives.

Extremely low learning rates (e.g., 0.00001) universally underperformed (AUROC <0.6; recall: 0.616–0.655; specificity: 0.451–0.518), indicating insufficient feature learning due to inadequate parameter updates within fixed training epochs. Performance improved markedly at learning rates of 0.00005–0.0001, though high learning rates (0.0003) introduced instability, evidenced by increased inter-batch variability (F1-score fluctuations of 0.012 between batch sizes 4 and 32) and declining specificity. Based on these findings, we selected a batch size of 16 and learning rate of 0.00005 for HyenaCircle, balancing predictive performance, training stability, and practical utility.

### 3.3 Long eccDNA prediction by HyenaCircle

In this work, we developed a base-resolution prediction algorithm for long eccDNA formation, HyenaCircle, by adapting the HyenaDNA large language model architecture to third-generation sequencing data and full-length eccDNA sequences. Following optimization of batch size and learning rate, the model was trained and validated. Evaluation results demonstrated the utility of HyenaCircle in predicting long eccDNA formation, achieving an AUROC of 0.715 on the validation set. Additional performance metrics included accuracy (0.670), precision (0.670), recall (0.776), specificity (0.545), and F1-score (0.719), indicating robust discriminative capability between positive and negative samples, with particular strength in detecting positive cases with 77.58% recall.

**FIGURE 3:**
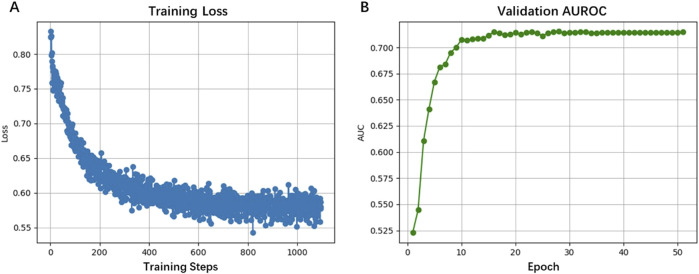
Performance of the HyenaCircle model on the training sets **(A)** and validation sets **(B)**.

Training curves in Figure 3A showed stable convergence, with loss values decreasing from 0.80 to 0.55 over 1,000 training steps (50 epochs). The validation AUROC displayed in Figure 3B progressively improved to 0.7147 during training, reflecting the model’s capacity to extract both global sequence features (up to 5 kb) and base-resolution patterns. This performance advantage stems from HyenaDNA’s specialized attention mechanism, which effectively captures long-range dependencies in DNA sequences, a recognized limitation of conventional approaches. Our findings suggest HyenaCircle offers a methodological advance for eccDNA identification and analysis.

### 3.4 Comparative experiments

**FIGURE 4:**
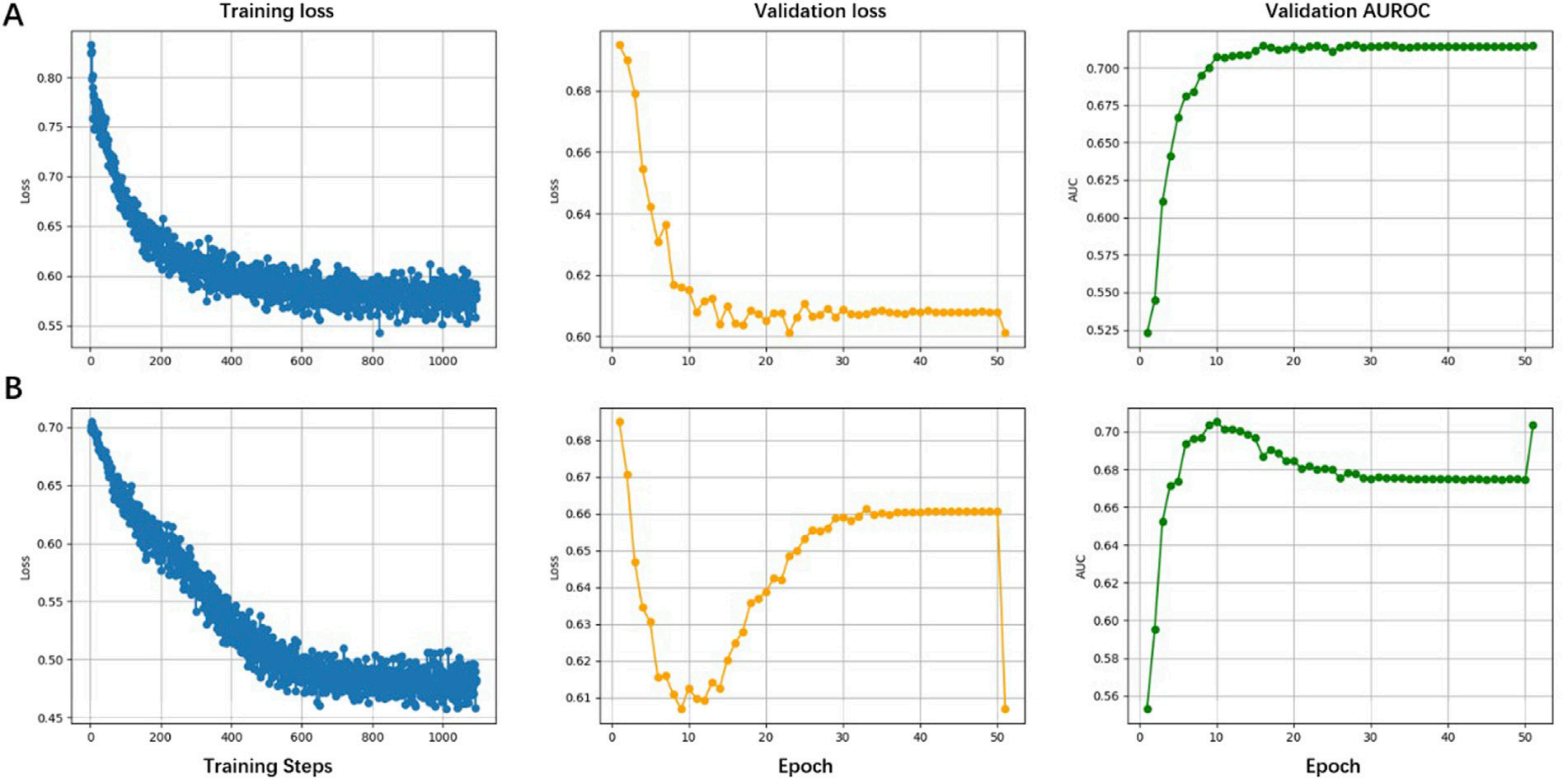
Comparison of the performance of the HyenaCircle model **(A)** and baseline models **(B)** on training and validation sets.

We further evaluated the predictive performance of HyenaCircle against the baseline hyenadna-small-32k model. Both models used identical batch sizes with 16, learning rates with 0.00005, and data preprocessing as well as data augmentation. As shown in Figure 4, HyenaCircle demonstrated superior training stability and predictive performance.

The training loss curves revealed that HyenaCircle reached stable convergence after 10 epochs, with final loss values below 0.6 (Figure 4A). In contrast, while the baseline model ultimately achieved lower loss values, it exhibited slower convergence, stabilizing only after 25 epochs, and greater instability during training. Validation loss curves indicated that HyenaCircle maintained stable generalization without overfitting, whereas the baseline model showed slight validation loss increases in later training phases (Figure 4B), suggesting potential overfitting risks. This implies that direct application of the baseline model, despite its strong feature representation capacity, may compromise generalizability without architectural modifications.

The observed improvements stem from HyenaCircle’s enhanced feature modeling capacity through strategic architectural additions to the baseline framework. This was further corroborated by validation AUROC curves, where HyenaCircle showed more stable and generalizable performance. Collectively, these results demonstrate that HyenaCircle achieves more efficient biological feature extraction for long eccDNA prediction compared to the baseline language model, even under identical data augmentation conditions.

To further validate the effectiveness of the HyenaCircle model, we conducted comparative experiments using the DNABERT model with parameters configured according to its original publication (Chang et al., 2023). The DNABERT model was employed by DeepCircle for predicting the formation of eccDNAs shorter than 1 kb; however, when applied to predict long eccDNA sequences (>1 kb), DNABERT achieved the following performance metrics on the test set: AUROC 0.6878, accuracy 0.637, precision 0.622, recall 0.739, specificity 0.529, and F1-score 0.675 (Table 3). The evaluation metrics indicate that DNABERT demonstrates limited overall accuracy in predicting long eccDNA sequences, failing to maintain the high performance reported in its original study for short-sequence prediction. While the model shows some discriminative capability (F1-score 0.6752, AUROC 0.6878) between positive and negative classes, its performance remains substantially lower than that of HyenaCircle. These comparative results highlight the superior suitability of the HyenaDNA architecture for long-sequence biological data analysis, particularly in eccDNA prediction tasks.

**TABLE 3:** Comparison of the model performance of DNABERT and HyenaCircle in predicting long eccDNA (1kb–5 kb).

Model	AUROC	Accuracy	Precision	Recall	Specificity	F1-score
DNABERT	0.675	0.637	0.622	0.739	0.529	0.675
HyenaCircle (Ours)	0.715	0.670	0.670	0.776	0.545	0.719

### 3.5 Assessment of flanking sequences on prediction performance

Given the importance of flanking sequences for eccDNA breakpoint identification, we systematically evaluated how truncating these regions impacts HyenaCircle’s predictive performance. Our results demonstrate that sequence context surrounding breakpoints critically influences model accuracy.

As shown in Table 4, the baseline model incorporating full flanking sequences achieved optimal performance, whose AUROC was 0.715 and F1-score was 0.719, and with balanced specificity (0.545) and recall (0.776). Truncation experiments revealed significant performance variations: while some conditions (e.g., 40 bp flanking sequences) showed improved recall (0.831), this came at the cost of markedly reduced specificity (minimum 0.463 at 60 bp), indicating compromised prediction stability. Systematic analysis showed that reduced sequence length decreased model specificity by an average of 9.7% compared to baseline, with recall and F1-scores fluctuating between 0.729–0.841 and 0.697–0.735 respectively, suggesting heightened sensitivity to local genomic features. The truncated models also exhibited lower discriminative capacity, with mean AUROC (0.711) consistently below baseline performance (0.715), accompanied by reduced accuracy and precision.

**TABLE 4:** Impact of upstream and downstream sequences of eccDNA breakpoint regions on the performance of the HyenaCircle model.

Length (bp)	AUROC	Accuracy	Precision	Recall	Specificity	F1-score
Baseline	0.715	0.670	0.670	0.776	0.545	0.719
10	0.709	0.666	0.660	0.795	0.512	0.721
20	0.711	0.671	0.657	0.825	0.488	0.732
30	0.712	0.667	0.667	0.776	0.538	0.717
40	0.714	0.675	0.659	0.831	0.488	0.735
50	0.708	0.671	0.659	0.817	0.498	0.730
60	0.704	0.668	0.651	0.841	0.463	0.734
70	0.717	0.671	0.672	0.772	0.552	0.719
80	0.705	0.663	0.667	0.759	0.550	0.710
90	0.707	0.656	0.669	0.729	0.570	0.697
100	0.713	0.668	0.669	0.769	0.547	0.716

These findings confirm that flanking sequences provide essential contextual information for eccDNA formation prediction. The performance degradation observed with truncated sequences aligns with our FLED algorithm’s design rationale, which similarly utilizes breakpoint-adjacent sequences for accurate detection.

## 4 Discussion

In this study, we addressed the challenging task of predicting long eccDNAs (1–5 kb) by developing the first deep learning framework that integrates full-length eccDNA sequences identified from third-generation sequencing (TGS) data with a large language model (LLM), systematically exploring the predictive value of genomic long-range dependencies in eccDNA formation.

Using FLED, we obtained 23,812 high-quality full-length eccDNA sequences from TGS data and incorporated 100 bp flanking genomic sequences surrounding eccDNA breakpoints as critical contextual information. At the model architecture level, we adapted the HyenaDNA framework and proposed HyenaCircle, which employs single-nucleotide tokenization, adaptive pooling, and a multi-layer perceptron (MLP)-based classification module to enable accurate prediction of 1–5 kb eccDNAs. Experimental results demonstrated that HyenaCircle significantly outperformed baseline models and DNABERT on the validation set, validating the superiority of HyenaDNA in long-sequence feature extraction.

Given the limited sample size in this study, we implemented systematic experimental designs to mitigate potential overfitting risks across dataset partitioning, preprocessing, model construction, and training. First, we split the dataset and determined optimal hyperparameters based on loss function curves in the training set before validating model performance on an independent internal test set. Additionally, we applied data augmentation techniques to the training set, including reverse-complement transformation, random truncation, and nucleotide substitution, ensuring that the introduced noise remained comparable to real sequencing errors. During model training, we employed dropout regularization to further prevent overfitting.

Furthermore, our study revealed the profound impact of hyperparameter tuning on model performance: a combination of moderate batch size and learning rate achieved a balance between gradient stochasticity and stability, yielding an F1-score of 0.719. Ablation experiments confirmed the importance of flanking genomic sequences near breakpoints, highlighting the biological relevance of structural features such as microhomologies in eccDNA formation.

Reliable detection of eccDNA typically relies on experimental validation. However, considering the cost of experimental validation and the applicability to large-scale samples, the positive samples in this study were derived from high-quality eccDNA sequences identified by FLED. Nevertheless, FLED depends on features such as split-read alignment patterns present in TGS sequencing data and may be affected by structural variation regions in linear DNA or alignment errors, thus posing a certain risk of false positives. Moreover, FLED imposes requirements on eccDNA size, abundance, and structural complexity; extremely large, highly complex, or low-abundance eccDNAs may not be reliably detected, and insufficient sequencing depth may also result in missed detections. Although FLED attempts to balance false-negative and false-positive risks as much as possible, some impact on HyenaCircle is inevitable, potentially limiting its predictive capability for eccDNA. HyenaCircle achieved only 67% precision and an AUROC of 0.71 on the validation set, indicating that the current model still requires further optimization in false-positive control and the extraction of specific sequence patterns, such as microhomology structures. Additionally, previous studies have suggested that eccDNA formation exhibits high stochasticity and low reproducibility, which may also contribute to the model’s performance limitations. In the future research, the establishment of high-confidence eccDNA database through experimental validation, along with the incorporation of error-correction modules for eccDNA detection into the HyenaCircle, will improve the predictive performance of HyenaCircle.

In this work, we demonstrate the predictability of long eccDNAs, which provides potential for their clinical application. First, sequence-based eccDNA formation prediction models may capture intrinsic sequence features underlying eccDNA biogenesis, thereby deepening understanding of tumor progression mechanisms. Second, eccDNA is an important mechanism driving resistance to targeted cancer therapies and is dynamically generated during tumor development. By analyzing genomic sequence features, such as specific amplification patterns and microhomology, it is possible to predict the propensity for eccDNA formation in tumors and even precancerous tissues. This can help identify patients exhibiting high tumor heterogeneity and potential resistance risk, classifying them into higher-risk categories and enabling the implementation of more proactive monitoring and therapeutic strategies. Furthermore, LLMs excel at capturing long-range dependencies and contextual information, allowing deep mining and quantitative characterization of eccDNA sequence features. In the future, integration of additional omics data, such as epigenomic or transcriptomic profiles, could enable construction of more comprehensive tumor diagnostic or prognostic models, thereby advancing precision oncology.

## 5 Conclusion

Overall, this work presents HyenaCircle, the first pretrained large language model specifically designed for long eccDNA from third-generation sequencing (TGS) data, achieving significant performance improvements over traditional methods, such as DNABERT, in predicting long eccDNA formation based solely on sequence information, establishing a SOTA benchmark. Furthermore, by extending sequences around eccDNA breakpoints and employing dynamic enhancement strategies, this study further validates the critical role of breakpoint-adjacent sequences in eccDNA biogenesis. From a data-driven perspective, we established connections with biological prior knowledge and systematically evaluated the contribution of long-range sequence dependencies in eccDNA prediction.

## Data Availability

Publicly available datasets were analyzed in this study. This data can be found here: The data is available at National Genomics Data Center (https://ngdc.cncb.ac.cn/) with the accession number HRA002605.

## References

[B1] Ambros I. M. Rumpler S. Luegmayr A. Hattinger C. M. Strehl S. Kovar H. (1997). Neuroblastoma cells can actively eliminate supernumerary MYCN gene copies by micronucleus formation--sign of tumour cell revertance? Eur. J. Cancer 33 (12), 2043–2049. 10.1016/s0959-8049(97)00204-9 9516850

[B2] An W. Guo Y. Bian Y. Ma H. Yang J. Li C. (2022). “MoDNA: motif-oriented pre-training for DNA language model,” in Proceedings of the 13th ACM international conference on bioinformatics, computational biology and health informatics. Northbrook, IL: Association for Computing Machinery.

[B3] An W. Guo Y. Bian Y. Ma H. Yang J. Li C. (2024). Advancing DNA language models through motif-oriented pre-training with MoDNA. BioMedInformatics 4 (2), 1556–1571. 10.3390/biomedinformatics4020085

[B4] Chang K. L. Chen J. H. Lin T. C. Leu J. Y. Kao C. F. Wong J. Y. (2023). Short human eccDNAs are predictable from sequences. Brief. Bioinform 24 (3), bbad147. 10.1093/bib/bbad147 37088981

[B5] Chapman O. S. Luebeck J. Sridhar S. Wong I. T. Dixit D. Wang S. (2023). Circular extrachromosomal DNA promotes tumor heterogeneity in high-risk medulloblastoma. Nat. Genet. 55 (12), 2189–2199. 10.1038/s41588-023-01551-3 37945900 PMC10703696

[B6] Chen Y. Qiu Q. She J. Yu J. (2023). Extrachromosomal circular DNA in colorectal cancer: biogenesis, function and potential as therapeutic target. Oncogene 42 (13), 941–951. 10.1038/s41388-023-02640-7 36859558 PMC10038807

[B7] Dillon L. W. Kumar P. Shibata Y. Wang Y. H. Willcox S. Griffith J. D. (2015). Production of extrachromosomal MicroDNAs is linked to mismatch repair pathways and transcriptional activity. Cell Rep. 11 (11), 1749–1759. 10.1016/j.celrep.2015.05.020 26051933 PMC4481157

[B8] Eugen-Olsen R. A. B. Hariprakash J. M. Oestergaard V. H. Regenberg B. (2025). Molecular mechanisms of extrachromosomal circular DNA formation. Nucleic Acids Res. 53 (5), gkaf122. 10.1093/nar/gkaf122 40037708 PMC11879418

[B9] Gaubatz J. W. (1990). Extrachromosomal circular DNAs and genomic sequence plasticity in eukaryotic cells. Mutat. Res. 237, 271–292. 10.1016/0921-8734(90)90009-g 2079966

[B10] Helmsauer K. Valieva M. E. Ali S. Chamorro González R. Schöpflin R. Röefzaad C. (2020). Enhancer hijacking determines extrachromosomal circular MYCN amplicon architecture in neuroblastoma. Nat. Commun. 11 (1), 5823. 10.1038/s41467-020-19452-y 33199677 PMC7669906

[B11] Ji Y. Zhou Z. Liu H. Davuluri R. V. (2021). DNABERT: pre-trained bidirectional encoder representations from transformers model for DNA-Language in genome. Bioinformatics 37 (15), 2112–2120. 10.1093/bioinformatics/btab083 33538820 PMC11025658

[B12] Jin J. Yu Y. Wang R. Zeng X. Pang C. Jiang Y. (2022). iDNA-ABF: multi-scale deep biological language learning model for the interpretable prediction of DNA methylations. Genome Biol. 23 (1), 219. 10.1186/s13059-022-02780-1 36253864 PMC9575223

[B13] Karami F. M. Karimfar N. Fazlollahpour Naghibi A. Shafa S. Ghasemi Shiran M. Ataei M. (2022). Revisiting characteristics of oncogenic extrachromosomal DNA as Mobile enhancers on neuroblastoma and glioma cancers. Cancer Cell Int. 22 (1), 200. 10.1186/s12935-022-02617-8 35614494 PMC9131661

[B14] Koche R. P. Rodriguez-Fos E. Helmsauer K. Burkert M. MacArthur I. C. Maag J. (2020). Extrachromosomal circular DNA drives oncogenic genome remodeling in neuroblastoma. Nat. Genet. 52 (1), 29–34. 10.1038/s41588-019-0547-z 31844324 PMC7008131

[B15] Le N. Q. K. Ho Q. T. Nguyen V. N. Chang J. S. (2022). BERT-Promoter: an improved sequence-based predictor of DNA promoter using BERT pre-trained model and SHAP feature selection. Comput. Biol. Chem. 99, 107732. 10.1016/j.compbiolchem.2022.107732 35863177

[B16] Li F. Ming W. Lu W. Wang Y. Li X. Dong X. (2023). FLED: a full-length eccDNA detector for long-reads sequencing data. Brief. Bioinform 24 (6), bbad388. 10.1093/bib/bbad388 37930031 PMC10632013

[B17] Li H. (2018). Minimap2: pairwise alignment for nucleotide sequences. Bioinformatics 34 (18), 3094–3100. 10.1093/bioinformatics/bty191 29750242 PMC6137996

[B18] Li H. (2021). New strategies to improve minimap2 alignment accuracy. Bioinformatics 37 (23), 4572–4574. 10.1093/bioinformatics/btab705 34623391 PMC8652018

[B19] Li H. Handsaker B. Wysoker A. Fennell T. Ruan J. Homer N. (2009). The sequence alignment/map format and SAMtools. Bioinformatics 25 (16), 2078–2079. 10.1093/bioinformatics/btp352 19505943 PMC2723002

[B20] Ling X. Han Y. Meng J. Zhong B. Chen J. Zhang H. (2021). Small extrachromosomal circular DNA (eccDNA): major functions in evolution and cancer. Mol. Cancer 20 (1), 113. 10.1186/s12943-021-01413-8 34479546 PMC8414719

[B21] Luo H. Chen C. Shan W. Ding P. Luo L. (2022). “iEnhancer-BERT: a novel transfer learning architecture based on DNA-language model for identifying enhancers and their strength,” Intelligent computing theories and application (Cham: Springer International Publishing).

[B22] Luo X. Zhang L. Cui J. An Q. Li H. Zhang Z. (2023). Small extrachromosomal circular DNAs as biomarkers for multi-cancer diagnosis and monitoring. Clin. Transl. Med. 13 (9), e1393. 10.1002/ctm2.1393 37649244 PMC10468585

[B23] Lv W. Pan X. Han P. Wang Z. Feng W. Xing X. (2022). Circle-seq reveals genomic and disease-specific hallmarks in urinary cell-free extrachromosomal circular DNAs. Clin. Transl. Med. 12 (4), e817. 10.1002/ctm2.817 35474296 PMC9042798

[B24] Møller H. D. Mohiyuddin M. Prada-Luengo I. Sailani M. R. Halling J. F. Plomgaard P. (2018). Circular DNA elements of chromosomal origin are common in healthy human somatic tissue. Nat. Commun. 9 (1), 1069. 10.1038/s41467-018-03369-8 29540679 PMC5852086

[B25] Morales C. García M. J. Ribas M. Miró R. Muñoz M. Caldas C. (2009). Dihydrofolate reductase amplification and sensitization to methotrexate of methotrexate-resistant Colon cancer cells. Mol. Cancer Ther. 8 (2), 424–432. 10.1158/1535-7163.MCT-08-0759 19190117

[B26] Morales C. Ribas M. Aiza G. Peinado M. A. (2005). Genetic determinants of methotrexate responsiveness and resistance in Colon cancer cells. Oncogene 24 (45), 6842–6847. 10.1038/sj.onc.1208834 16007155

[B27] Morton A. R. Dogan-Artun N. Faber Z. J. MacLeod G. Bartels C. F. Piazza M. S. (2019). Functional enhancers shape extrachromosomal oncogene amplifications. Cell 179 (6), 1330–41.e13. 10.1016/j.cell.2019.10.039 31761532 PMC7241652

[B28] Nathanson D. A. Gini B. Mottahedeh J. Visnyei K. Koga T. Gomez G. (2014). Targeted therapy resistance mediated by dynamic regulation of extrachromosomal mutant EGFR DNA. Science 343 (6166), 72–76. 10.1126/science.1241328 24310612 PMC4049335

[B29] Nguyen E. Poli M. Faizi M. Thomas A. Birch-Sykes C. Wornow M. (2023). HyenaDNA: long-range genomic sequence modeling at single nucleotide resolution. ArXiv, arXiv:2306.15794v2.

[B30] Nikolaev S. Santoni F. Garieri M. Makrythanasis P. Falconnet E. Guipponi M. (2014). Extrachromosomal driver mutations in glioblastoma and low-grade glioma. Nat. Commun. 5, 5690. 10.1038/ncomms6690 25471132 PMC4338529

[B31] Noer J. B. Hørsdal O. K. Xiang X. Luo Y. Regenberg B. (2022). Extrachromosomal circular DNA in cancer: history, current knowledge, and methods. Trends Genet. 38 (7), 766–781. 10.1016/j.tig.2022.02.007 35277298

[B32] Ooi A. Takehana T. Li X. Suzuki S. Kunitomo K. Iino H. (2004). Protein overexpression and gene amplification of HER-2 and EGFR in colorectal cancers: an immunohistochemical and fluorescent *in situ* hybridization study. Mod. Pathol. 17 (8), 895–904. 10.1038/modpathol.3800137 15143334

[B33] Paulsen T. Kumar P. Koseoglu M. M. Dutta A. (2018). Discoveries of extrachromosomal circles of DNA in normal and tumor cells. 10.1016/j.tig.2017.12.010 PMC5881399 29329720

[B34] Paulsen T. Malapati P. Shibata Y. Wilson B. Eki R. Benamar M. (2021). MicroDNA levels are dependent on MMEJ, repressed by c-NHEJ pathway, and stimulated by DNA damage. Nucleic Acids Res. 49 (20), 11787–11799. 10.1093/nar/gkab984 34718766 PMC8599734

[B35] Prada-Luengo I. Krogh A. Maretty L. Regenberg B. (2019). Sensitive detection of circular DNAs at single-nucleotide resolution using guided realignment of partially aligned reads. BMC Bioinforma. 20 (1), 663. 10.1186/s12859-019-3160-3 PMC6909605 31830908

[B36] Prada-Luengo I. Møller H. D. Henriksen R. A. Gao Q. Larsen C. E. Alizadeh S. (2020). Replicative aging is associated with loss of genetic heterogeneity from extrachromosomal circular DNA in *Saccharomyces cerevisiae* . Nucleic Acids Res. 48 (14), 7883–7898. 10.1093/nar/gkaa545 32609810 PMC7430651

[B37] Quinlan A. R. (2014). BEDTools: the swiss-army tool for genome feature analysis. Curr. Protoc. Bioinforma. 47 (11.2), 11.12.1–11.12.34. 10.1002/0471250953.bi1112s47 PMC4213956 25199790

[B38] Quinlan A. R. Hall I. M. (2010). BEDTools: a flexible suite of utilities for comparing genomic features. Bioinformatics 26 (6), 841–842. 10.1093/bioinformatics/btq033 20110278 PMC2832824

[B39] Shibata Y. Kumar P. Layer R. Willcox S. Gagan J. R. Griffith J. D. (2012). Extrachromosomal microDNAs and chromosomal microdeletions in normal tissues. Science. 336 (6077), 82–86. 10.1126/science.1213307 22403181 PMC3703515

[B40] Tsukiyama S. Hasan M. M. Deng H. W. Kurata H. (2022). BERT6mA: prediction of DNA N6-methyladenine site using deep learning-based approaches. Brief. Bioinform 23 (2), bbac053. 10.1093/bib/bbac053 35225328 PMC8921755

[B41] Wang S. Wu C. Y. He M. M. Yong J. X. Chen Y. X. Qian L. M. (2024). Machine learning-based extrachromosomal DNA identification in large-scale cohorts reveals its clinical implications in cancer. Nat. Commun. 15 (1), 1515. 10.1038/s41467-024-45479-6 38373991 PMC10876971

[B42] Wang Y. Wang M. Djekidel M. N. Chen H. Liu D. Alt F. W. (2021). eccDNAs are apoptotic products with high innate immunostimulatory activity. Nature 599 (7884), 308–314. 10.1038/s41586-021-04009-w 34671165 PMC9295135

[B43] Wang Y. A.-O. Wang M. A.-O. Zhang Y. A.-O. (2023). Purification, full-length sequencing and genomic origin mapping of eccDNA. 10.1038/s41596-022-00783-7 36517607

[B44] Wu S. Turner K. M. Nguyen N. Raviram R. Erb M. Santini J. (2019). Circular ecDNA promotes accessible chromatin and high oncogene expression. Nature 575 (7784), 699–703. 10.1038/s41586-019-1763-5 31748743 PMC7094777

[B45] Yang L. Jia R. Ge T. Ge S. Zhuang A. Chai P. (2022). Extrachromosomal circular DNA: biogenesis, structure, functions and diseases. Signal Transduct. Target Ther. 7 (1), 342. 10.1038/s41392-022-01176-8 36184613 PMC9527254

[B46] Yang Y. Song T. Liu S. Liu Z. Wang X. Li Y. (2024). Circle-map profiling of extrachromosomal circular DNA as diagnostic biomarkers for lung cancer. Precis. Clin. Med. 7 (1), pbae006. 10.1093/pcmedi/pbae006 38616889 PMC11015151

[B47] Zhu Y. Gujar A. D. Wong C. H. Tjong H. Ngan C. Y. Gong L. (2021). Oncogenic extrachromosomal DNA functions as Mobile enhancers to globally amplify chromosomal transcription. Cancer Cell 39 (5), 694–707.e7. 10.1016/j.ccell.2021.03.006 33836152 PMC8119378

